# Gut microbiota modulation in the prevention and treatment of heat stroke

**DOI:** 10.3389/fimmu.2026.1837970

**Published:** 2026-06-02

**Authors:** Zhenglian Wang, Jun Yan, Jing Xiao, Weijun Guo, Maolin Deng

**Affiliations:** Department of Emergency, Changsha Hospital of Traditional Chinese Medicine, Changsha, China

**Keywords:** gut microbiota, heat stroke, intestinal barrier, multiple organ dysfunction, systemic inflammatory response syndrome

## Abstract

In recent years, heat stroke (HS) have been reported with increasing frequency, and this trend is hard to separate from broader environmental changes, including climate change, recurrent extreme heat events, and air pollution. When people are exposed to high-temperature environments for a prolonged period, especially during intense physical activity, the condition may progress to HS. HS is an acute and potentially fatal disorder that can deteriorate rapidly if not treated in time. The intestine appears to be particularly vulnerable during HS. HS can disrupt intestinal tight junctions and weaken the barrier function of the gut, leading to what is commonly described as a “leaky gut.” Once this barrier is compromised, microbial products such as lipopolysaccharides can enter the bloodstream. These molecules may then activate immune cells, promote excessive cytokine release, and eventually drive a systemic inflammatory response. In severe cases, this inflammatory process can develop into systemic inflammatory response syndrome (SIRS) and multiple organ dysfunction syndrome (MODS). Current evidence increasingly suggests that intestinal injury is not simply a secondary result of HS. Rather, it may serve as an important early event in the development and progression of the disease. Still, it should be noted that much of the available evidence comes from preclinical studies, and strong clinical confirmation is still limited. Probiotics have attracted attention because they may help reduce the occurrence and severity of HS by maintaining gut microbiota balance and regulating intestinal immune responses. However, since most supporting data are still derived from animal experiments, their protective effects in humans need to be interpreted carefully. Another point worth emphasizing is that the gut is not working alone. Through gut–organ communication networks, the intestinal microbiota can interact with distant organs, including the liver, lungs, and brain. These gut–liver, gut–lung, and gut–brain axes may help explain how HS leads to injury beyond the intestine itself. In this review, we summarize current findings on how modulation of the gut microbiota may improve intestinal thermotolerance and strengthen barrier function, with the aim of providing useful insights for the prevention and treatment of HS in clinical practice.

## 1 Introduction

Heat stroke (HS) is the most severe form of heat-related illness, characterized by a rapid elevation of core body temperature, central nervous system dysfunction, and multiple organ failure. It has a sudden onset, rapid progression, and a high mortality rate (1, 2). Based on etiology, HS is classified into classic heat stroke (CHS) and exertional heat stroke (EHS) (3). In recent years, the incidence of HS has been steadily increasing due to global climate change and reduced human heat tolerance. According to the Lancet 2024 report, the average global exposure to high temperatures in 2023 was 50 days higher than expected, reaching a record high (4), posing significant challenges to HS prevention and treatment.

Intestinal injury is a critical initiating factor in the pathogenesis of HS. Under high-temperature conditions, oxidative stress disrupts the intestinal mucosal structure, increases intestinal permeability, and leads to “leaky gut,” facilitating the translocation of endotoxins and pathogens into the bloodstream. Concurrently, alterations in the composition and abundance of the gut microbiota affect nutrient absorption and local immune homeostasis (5–7). Studies have shown that heat stress disrupts tight junctions, activates immune cells, and triggers a cytokine storm, ultimately resulting in systemic inflammatory response syndrome (SIRS) and multiple organ dysfunction (MODS) (8, 9) Microbial products can also cross the blood–brain barrier (BBB) to induce neuroinflammation, and intestinal necrosis with hemorrhage as well as activation of the extrinsic coagulation pathway are frequently observed in fatal cases (10–12).

**Figure 1:**
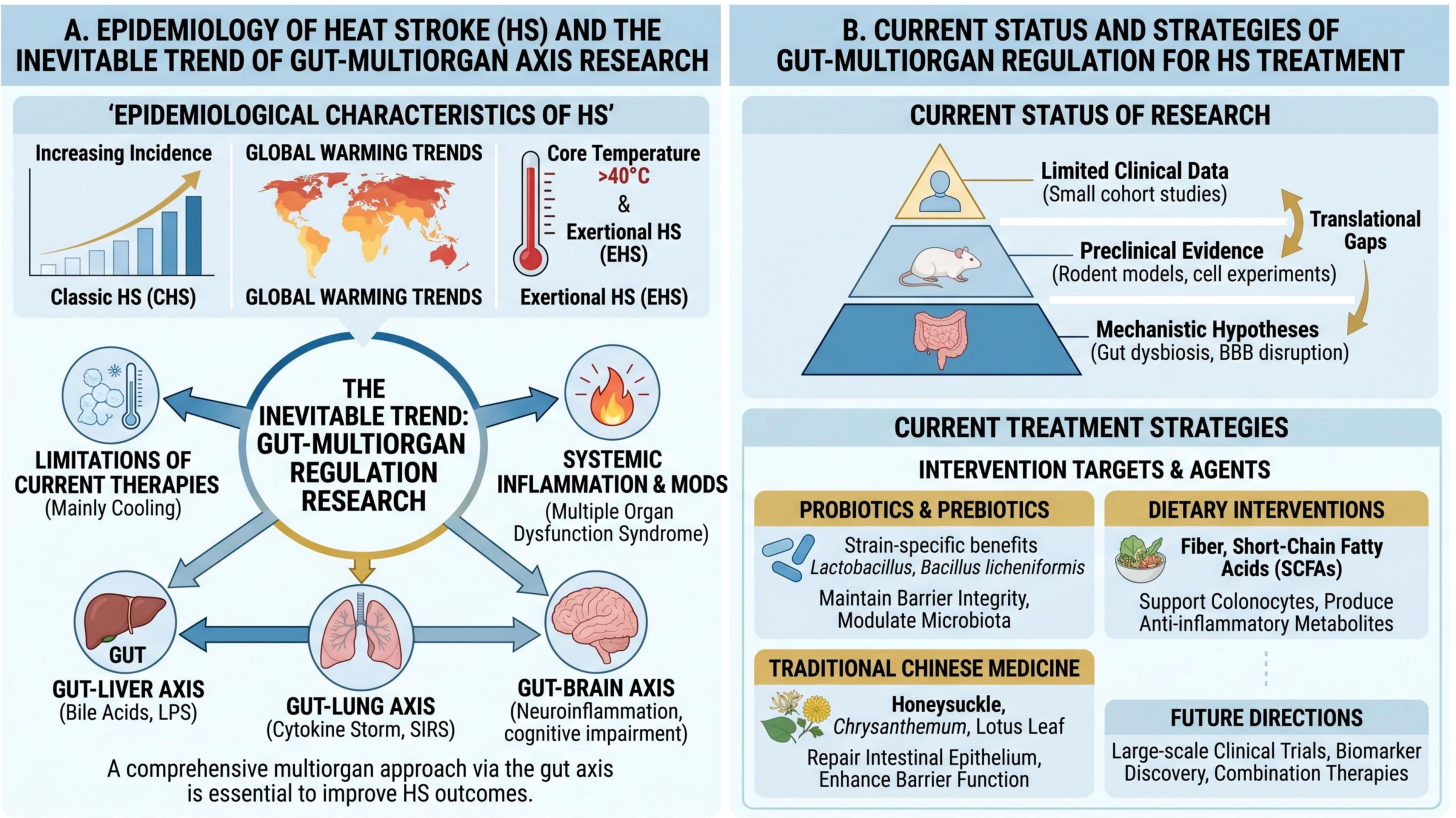
Epidemiological background, current research status, and therapeutic strategies targeting the gut–multiorgan axis in heat stroke. **(A)** With global warming and the increasing frequency of extreme heat events, the risk of heat stroke (HS) continues to rise. Both classic heat stroke and exertional heat stroke may present with a core body temperature exceeding 40 °C, subsequently triggering systemic inflammatory responses and multiple organ dysfunction. Current clinical management still relies primarily on rapid cooling, whereas interventions targeting intestinal barrier disruption, endotoxin translocation, and secondary multiorgan injury remain limited. The gut may participate in bile acid and lipopolysaccharide transport, cytokine storm, neuroinflammation, and cognitive impairment through the gut–liver, gut–lung, and gut–brain axes. Therefore, gut–multiorgan regulation is increasingly recognized as an important direction in HS research. **(B)** Existing studies indicate that research on gut-related regulation in HS still faces challenges, including insufficient clinical evidence and limited translational application. At the mechanistic level, current investigations mainly focus on gut microbiota dysbiosis, intestinal mucosal barrier injury, and blood–brain barrier disruption, with most evidence derived from rodent models and cell-based experiments. Potential intervention strategies include probiotics/prebiotics, dietary fiber and short-chain fatty acids, as well as traditional Chinese medicine. These approaches may act by maintaining intestinal barrier integrity, modulating gut microbiota composition, promoting the production of anti-inflammatory metabolites, and repairing the intestinal epithelial barrier. Future studies are needed to validate their clinical value through large-scale clinical trials, biomarker discovery, and combined therapeutic strategies.

Heat-induced gut dysbiosis is characterized by a reduction in beneficial bacteria such as *Lactobacillus* and *Bifidobacterium*, accompanied by the overgrowth of opportunistic pathogens (13). Although current evidence remains limited, hyperthermia may also affect the fungal microbiota; however, fungal dysbiosis in HS is still insufficiently characterized and should be regarded as a hypothesis requiring further investigation (12, 14, 15). Moreover, most available data are derived from animal or experimental models, suggesting that heat exposure may perturb the fungal microbiota and thereby aggravate mucosal inflammation, although the clinical relevance of this observation in HS remains unclear and requires further validation. In addition, heat-induced microbial translocation may initiate a persistent gut–endothelium–brain inflammatory axis: circulating lipopolysaccharide (LPS) and damage-associated molecular patterns (DAMPs), such as HMGB1, can bind to Toll-like receptor 4, activate an NF-κB-dependent cytokine cascade, and disrupt the blood–brain barrier (BBB), thereby contributing to sustained central nervous system dysfunction (16, 17). Recent studies further suggest that gut-derived inflammatory mediators and dysbiosis may persist during the recovery phase, leading to long-term cognitive and motor impairments in survivors (2, 18–20). Factors such as repeated heat exposure, aging, and environmental pollution may further exacerbate intestinal permeability and immune dysfunction (19, 20) (**Figure 1**). Notably, much of the evidence supporting the involvement of the gut microbiota in HS is derived from rodent models. Therefore, the translational relevance of these findings to humans should be interpreted with caution, particularly regarding differences in microbiota composition, heat tolerance, dose exposure, and the timing of intervention.

These findings highlight the intestinal epithelial–microbiota axis as a critical target for early intervention in HS. Although early cooling remains the primary treatment for HS, the mortality rate among severe cases remains high, underscoring the urgent need for effective preventive strategies. Studies have shown that probiotics can enhance intestinal barrier function and reduce the risk of HS by modulating the gut microbiota (21). This review summarizes recent advances in the regulation of the gut microbiota for the prevention and treatment of HS, aiming to provide insights for the development of preventive strategies and optimization of clinical management.

## 2 Key role of the gut microbiota in the pathogenesis of HS

### 2.1 Gut dysbiosis and intestinal barrier injury in the pathogenesis of HS

The gastrointestinal tract represents the largest interface between the body and the external environment (22). The intestinal epithelium serves as a physical and biochemical barrier that separates the gut microbiota from the mucosal immune system (11). Comprising over 1,000 microbial species—including bacteria, viruses, fungi, and parasites—the gut microbiota plays a critical role in regulating host nutrient metabolism and maintaining immune homeostasis (23, 24). Changes in ambient temperature can induce alterations in the intestinal microenvironment, leading to microbial remodeling, a process closely associated with various physiological and pathological mechanisms (25).

Studies have shown that exposure to humid heat induces gut dysbiosis in HS mice, primarily characterized by a reduced abundance of beneficial bacteria such as *Lactobacillus murinus*, which in turn impairs intestinal barrier function (26, 27). HS-induced structural changes in the gut microbiota increase intestinal permeability and promote bacterial translocation via the portal vein and mesenteric lymphatics, contributing to systemic infection and distant organ injury (23). Therefore, maintaining gut microbiota homeostasis is of great significance for the prevention and treatment of HS: from a preventive perspective, it reduces the risk of systemic infection by enhancing barrier function; from a therapeutic perspective, it facilitates recovery of intestinal function by alleviating inflammation and improving metabolic disturbances.

Microbial 16S rRNA sequencing analysis has revealed that humid heat exposure leads to gut dysbiosis, manifested as a decrease in *Lactobacillus murinus* abundance, while metabolomic analysis shows elevated serum levels of secondary bile acids (e.g., lithocholic acid) (25). Concurrently, increased expression of pro-inflammatory cytokines in the serum and cerebral cortex, activation of the PI3K/AKT/NF-κB signaling pathway, and microglial activation in the cortex have been observed, indicating enhanced neuroinflammatory responses (25). Transplantation of the gut microbiota from humid heat-exposed mice into germ-free mice recapitulated these abnormalities, whereas supplementation with *Lactobacillus murinus* significantly reversed them. These findings suggest that humid heat-induced gut dysbiosis drives disease progression by interfering with bile acid metabolism and exacerbating neuroinflammation, with probiotic intervention representing a potential therapeutic strategy.

HS may reshape the intestinal ecosystem through multiple converging mechanisms rather than a single pathway. First, reduced splanchnic perfusion and epithelial hypoxia can compromise mucus production and tight-junction integrity, creating a permissive niche for pathobionts. Second, heat-induced oxidative stress and mitochondrial dysfunction may alter epithelial antimicrobial defense and favor inflammatory taxa. Third, changes in feed intake, hydration status, bile acid circulation, and intestinal transit can modify luminal substrates, thereby affecting microbial composition and function. These events are often accompanied by depletion of short-chain fatty acid-producing bacteria and enrichment of opportunistic organisms, which together reduce epithelial energy supply and weaken barrier resilience.

Among the microbial mediators implicated in HS, SCFAs appear to be particularly important because they support colonocyte metabolism, enhance mucus production, and promote regulatory immune responses (27, 28). By contrast, reduced bile acid homeostasis may impair FXR/TGR5 signaling and aggravate epithelial injury and inflammation (29, 30). Tryptophan-derived metabolites may further modulate the aryl hydrocarbon receptor pathway and shape mucosal immune tolerance. At the signaling level, microbial products such as lipopolysaccharide can activate TLR4/NF-κB signaling, while cellular stress and mitochondrial injury may promote inflammasome activation, including NLRP3. Together, these pathways form a feed-forward loop linking heat stress, dysbiosis, barrier failure, and systemic inflammatory amplification.

### 2.2 Central role of intestinal barrier injury in the pathogenesis of HS

The intestinal barrier, composed of intestinal epithelial cells and tight junctions, exhibits selective permeability and is essential for maintaining overall health (31, 32). During HS, the intestine is more sensitive to heat stress than the liver, and barrier dysfunction can occur at an early stage (33) (**Figure 1A**). Hematoxylin and eosin staining of intestinal tissues from EHS mice reveals marked injury, characterized by villous enlargement and deformation, lamina propria separation, capillary dilation, and increased cellular infiltration within the lamina propria (34). Barrier disruption further induces microbial translocation, activates inflammatory cascades, and triggers endotoxemia, ultimately leading to systemic inflammatory response syndrome (SIRS) and subsequent MODS, creating a secondary insult to the intestine (35, 36).

Maintaining intestinal barrier integrity is critical for HS prevention and treatment (37). Traditional Chinese medicines such as *Lonicerae japonicae flos* (honeysuckle), *Chrysanthemi flos* (chrysanthemum), and *Nelumbinis folium* (lotus leaf) have been shown to enhance or repair intestinal barrier function (38). Probiotic formulations can promote intestinal epithelial cell healing and protect barrier integrity (39). Li et al. (26) reported that *Bacillus licheniformis*, a probiotic strain, alleviated tissue injury in HS rats by preserving intestinal barrier function and modulating the gut microbiota, thereby providing a novel intervention target for HS management (**Figure 1A**).

Overall, the available evidence supporting gut microbiota-based interventions in HS should be interpreted according to study design and level of validation. Most findings are derived from cell-based experiments and animal models, whereas only limited human data are currently available. In general, probiotics and dietary interventions showing benefit in rodents are promising, but their efficacy, optimal dose, timing, and strain specificity in humans remain uncertain. Therefore, conclusions drawn from preclinical studies should be considered hypothesis-generating rather than practice-changing (**Figure 1B**).

## 3 Exploring the role of gut microbiota modulation in HS prevention and treatment via the gut–liver/gut-lung/gut-brain axis

Existing evidence suggests that, during HS, the intestine represents an early and critical target organ of heat stress (40). Hyperthermia, together with ischemia and hypoxia, can disrupt intestinal epithelial tight junctions, increase intestinal permeability, and facilitate the translocation of bacterial components and endotoxins into the circulation, thereby triggering a sepsis-like systemic inflammatory response and multiple organ injury (41). Within this context, the gut–liver axis provides a mechanistic framework for explaining how portal vein-derived lipopolysaccharide, dysregulated bile acid metabolism, reduced short-chain fatty acids, and *Kupffer* cell activation may exacerbate liver injury as well as coagulation and metabolic abnormalities (42, 43). Similarly, the gut–lung axis may account for HS-associated acute lung injury, disruption of the alveolar–capillary barrier, and increased susceptibility to secondary infection through alterations in microbial metabolites, mucosal immunity, and the migration of inflammatory mediators (44). The gut–brain axis is also closely associated with post-HS disturbances of consciousness, cognitive impairment, and neuroinflammation (45).

Recent animal studies further support this research direction. In models of exertional heat stroke, alterations in the gut microbiota and metabolic profiles have been observed, particularly a reduction in *Lactobacillaceae/Lactobacillus murinus*. Supplementation with *Lactobacilli* has been shown to alleviate cognitive impairment in mice, potentially through upregulation of the hippocampal BDNF/TrkB pathway, suggesting that targeting the gut microbiota may not only improve intestinal barrier function but also influence central nervous system outcomes (34, 46).

Compared with the existing literature, which has largely focused on isolated aspects—such as intestinal barrier injury, gut-derived endotoxemia, the protective effects of probiotics, or the gut–brain axis alone—the distinctive feature of this section is that it proposes the gut microbiota as a shared upstream regulatory hub (47). On this basis, HS-related liver, lung, and brain injuries are integrated into a unified “gut–multi-organ axis” framework (48). This approach enables a systematic comparison of the causal links among alterations in microbial community structure, changes in microbial metabolites, immune-inflammatory pathways, barrier function, and organ-specific injury. It also allows further evaluation of the feasibility of interventions such as probiotics/prebiotics, postbiotics, dietary modulation, fecal microbiota transplantation, and regulation of short-chain fatty acids or bile acids for the prevention and treatment of HS.

The potential future significance of this framework is twofold. First, it may promote the expansion of HS risk prediction beyond conventional indicators such as core body temperature and inflammatory markers to include microbial biomarkers, indices of intestinal permeability, and metabolomic signatures. Second, it may provide earlier and more individualized adjunctive intervention strategies beyond the current paradigm of “cooling plus organ support,” with particular relevance for preventive management in high-temperature occupational settings, military training, sports medicine, and elderly populations with chronic diseases.

### 3.1 Gut–liver axis in HS

The gut–liver axis serves as a bidirectional communication system between the intestine and the liver and plays a critical role in the pathogenesis and management of HS (49). Regarding disease progression, heat stress during HS induces gut dysbiosis, characterized by a reduction in beneficial bacteria, proliferation of pathogenic microorganisms, and an altered *Firmicutes/Bacteroidetes* ratio (50). This dysbiosis disrupts intestinal barrier integrity, leading to endotoxin translocation into the systemic circulation and subsequent activation of hepatic immune responses, manifested by increased release of pro-inflammatory cytokines such as IL-1β and upregulation of acute-phase proteins such as orosomucoid 2 (ORM2). Elevated ORM2 levels, a liver-derived protein, positively correlate with the degree of gut dysbiosis, suggesting that the liver can sense and respond to inflammation signals originating from the intestine (51) (**Figure 1**). Additionally, gut microbial metabolites—including SCFAs and bile acids—are transported to the liver via the portal vein, where they participate in the regulation of hepatocyte membrane phospholipid synthesis, energy metabolism, and inflammatory responses. Disruption of these metabolic pathways may further exacerbate liver injury (52).

From an intervention perspective, several gut–liver axis–related strategies appear to be worth attention. Baicalin, for example, has been reported to alleviate heat stress-induced liver injury, probably by strengthening intestinal barrier function and reshaping the composition of the cecal microbiota (53). Tea polysaccharides may also be beneficial. Rather than acting only on the liver itself, they seem to suppress hepatic inflammation by altering inflammation-associated gut microbial populations and modulating serum long-chain fatty acid profiles. These findings, in a way, remind us that liver protection during heat stress may begin quite early at the intestinal level. The liver is not merely a passive target in this process, either. Pigment epithelium-derived factor (PEDF), a liver-derived factor, helps maintain intestinal stem cell homeostasis through the Wnt/β-catenin signaling pathway. Interestingly, when intestinal inflammation is detected, the liver can downregulate PEDF expression via PPAR-α signaling, which may help speed up intestinal tissue repair (54). In parallel, gut microbiota-derived short-chain fatty acids (SCFAs) can promote hepatic membrane phospholipid synthesis and support hepatocyte regeneration (55). So, the communication between the gut and liver is clearly not one-way; it is more like a dynamic feedback loop. Taken together, these studies suggest that targeting the gut–liver axis may help reduce HS-associated liver injury and systemic inflammation. Potential mechanisms include preservation of intestinal barrier integrity, remodeling of gut microbiota composition, maintenance of bile acid metabolic balance, and regulation of liver-derived protective factors. That said, the field is still not fully settled. More work is needed to clarify exactly how probiotics and other microbiota-oriented interventions protect against HS through the gut–liver axis, especially before these findings can be confidently translated into clinical practice.

For the gut–liver axis, most mechanistic data derive from experimental heat stress models, with limited clinical validation. The proposed role of bacterial translocation, endotoxemia, and hepatic inflammatory signaling in HS is biologically plausible, but direct human evidence remains sparse.

### 3.2 Gut–lung axis

The gut–lung axis refers to the bidirectional communication between the intestine and the lungs. In the context of heat stroke (HS), heat stress-induced intestinal hypoperfusion and barrier disruption may increase gut permeability and facilitate bacterial translocation, thereby potentially contributing to pulmonary complications such as acute lung injury (ALI) or acute respiratory distress syndrome (ARDS) (56, 57). However, direct evidence linking the gut–lung axis to HS remains limited, and much of the current understanding is extrapolated from non-HS models, including sepsis- and ALI/ARDS-related studies.

Mechanistically, evidence from sepsis models suggests that memory γδ T17 cells originating from the small intestine can migrate to the lungs via Wnt/β-catenin signaling and produce pro-inflammatory mediators such as interleukin-17A (IL-17A), thereby aggravating pulmonary inflammation (58). Likewise, in mouse models of ALI/ARDS, alterations in both intestinal and pulmonary microbiota have been observed, including reductions in *Firmicutes* and increases in *Actinobacteria*, suggesting that dysbiosis may participate in lung injury and may correlate with disease severity (59) (**Figure 1**). These findings support the concept that gut microbiota disturbances may influence lung pathology through immune and microbial crosstalk, although their relevance to HS requires further validation.

With regard to intervention, heat stress-induced upregulation of HSP70 has been reported to exert cytoprotective effects, but it may also be associated with pulmonary inflammatory responses. In addition, studies in asthma and other inflammatory lung diseases have shown that gut microbes, such as *Helicobacter pylori*, may modulate HSP70 expression through gut–lung communication and thereby influence airway inflammation (60, 61). Microbial metabolites, particularly SCFAs, are known to contribute to immune homeostasis in both the gut and the lungs, and experimental studies have indicated that microbiota-targeted interventions can ameliorate lung injury (62, 63). For example, *Akkermansia muciniphila* has been shown in non-HS models to attenuate lung tissue damage, reduce LPS-induced oxidative stress and inflammatory responses, and preserve intestinal barrier integrity through suppression of TLR2/MyD88/NF-κB signaling. Similarly, butyrate supplementation has been reported to alleviate histopathological injury and inflammatory responses in ALI mice, while restoring intestinal butyrate levels (64). Overall, these findings suggest that maintaining gut microbiota homeostasis and regulating microbial metabolites may represent a potential strategy for mitigating HS-associated lung injury; however, direct confirmation in HS-specific models and clinical cohorts is still lacking.

For the gut–lung axis, several conclusions are based on pulmonary inflammatory diseases rather than HS-specific studies. Thus, while dysbiosis may contribute to systemic inflammation and lung injury during HS, the causal relationship has not yet been firmly established.

### 3.3 Gut–brain axis

Neuroinflammation is widely recognized as one of the major drivers of central nervous system injury and poor neurological outcomes in HS (20). As HS progresses, heat stress can damage the nervous system through several overlapping mechanisms rather than through a single linear pathway (9). One important route involves systemic inflammatory response syndrome (SIRS). During this process, damage-associated molecular patterns (DAMPs), such as HMGB1 and extracellular histones, are released and subsequently activate Toll-like receptors and inflammasomes. This, in turn, promotes the production of pro-inflammatory cytokines, including IL-1β, IL-6, and TNF-α. Once this inflammatory cascade is set in motion, it can compromise the integrity of the blood–brain barrier (BBB), activate glial cells, and further intensify neuroinflammation and neuronal apoptosis (20, 65, 66). At the same time, hyperthermia itself should not be overlooked. Excessive heat can directly induce oxidative stress, impair mitochondrial function, increase the accumulation of reactive oxygen species, and ultimately contribute to neuronal cell death (67). Beyond these relatively well-described inflammatory and oxidative pathways, the gut–brain axis also appears to play a meaningful role. Gut microbiota and their metabolites may participate in the regulation of neuroinflammation during HS. For instance, exposure to humid heat can disturb gut microbial homeostasis, disrupt bile acid metabolism, and increase the permeability of both the intestinal barrier and the BBB. These changes may then promote neuroinflammatory responses and alter neurotransmitter-related signaling, eventually contributing to anxiety-like behaviors and cognitive impairment (25). Clinically, this is not just a theoretical concern. Available data suggest that approximately 10–28% of patients with HS experience persistent cognitive or motor dysfunction after the acute phase. Such long-term neurological sequelae have been linked to heat injury-induced gut dysbiosis, metabolic abnormalities, and reduced expression of brain-derived neurotrophic factor (BDNF) in the hippocampus. Taken together, these findings suggest that neurological injury in HS is shaped by a complex interaction among systemic inflammation, direct heat-induced cellular damage, and gut–brain axis dysfunction.

In terms of intervention strategies, probiotics have demonstrated potential protective effects. Lactobacillus rhamnosus reverses humid heat-induced gut dysbiosis, reduces serum secondary bile acid levels, and alleviates neuroinflammation and anxiety-like behavior (25). Probiotic supplementation upregulates hippocampal BDNF expression via the gut–brain axis, activates associated signaling pathways, supports neuroplasticity, and ameliorates cognitive impairment in EHS mice (34). Metabolite-based interventions also hold promise: β-hydroxybutyrate (BHBA) alleviates HS-induced neurological damage by regulating gut microbial homeostasis, suppressing endoplasmic reticulum stress (ERS), and mitigating microglial neuroinflammation (68). Protective factors such as the anti-inflammatory cytokine IL-10, heat shock proteins, and IL-1 receptor antagonists have also shown anti-inflammatory effects, while mesenchymal stem cells exert neuroprotective effects by reducing inflammation and preserving brain tissue structure. Collectively, these findings suggest that targeting the gut–brain axis by maintaining gut microbiota homeostasis, modulating microbial metabolites, suppressing neuroinflammation, and preserving BBB integrity can effectively alleviate HS-associated neurological injury.

For the gut–brain axis, evidence suggests that microbial metabolites, circulating endotoxins, and inflammatory mediators may influence BBB integrity and neuroinflammation. However, current support is largely derived from animal models, and whether the same mechanisms operate in human HS requires further investigation.

## 4 Therapeutic modulation of gut microbiota in HS

At present, studies on microbiota-targeted interventions for HS can be roughly viewed in three layers, though the boundaries are not always perfectly clear. The first layer is the most directly relevant evidence: findings from HS animal models, together with a small number of observational clinical studies. This is the evidence that speaks most closely to HS itself. The second layer is more indirect. It comes from related disease settings—such as sepsis, inflammatory bowel disease, pneumonia, asthma, and metabolic disorders. These studies can help explain possible mechanisms and make the biological rationale more convincing, but, strictly speaking, they should not be treated as HS-specific evidence. Then there is a third layer, which is more exploratory in nature. This includes *in vitro* experiments and small pilot studies. They are useful for pointing out potential targets or giving researchers a direction to follow, but they are still far from enough to support clinical recommendations. Keeping this three-tier framework in mind is especially important when interpreting studies on probiotic strains, traditional Chinese medicine (TCM) formulas, and microbiota-derived metabolites. After all, their effects are rarely one-size-fits-all. They can vary quite a bit depending on the specific strain, the disease context, when the intervention is given, and the background of the host. In other words, promising results in one setting do not automatically mean the same approach will work in HS.

From a translational perspective, microbiota-targeted interventions should not be considered substitutes for rapid cooling and organ support, which remain the cornerstone of HS management (69). Rather, they may serve as preventive adjuncts before heat exposure, early protective measures during the initial phase of HS, or supportive therapies after diagnosis once intestinal barrier disruption and systemic inflammation have been established (70). Nevertheless, important translational barriers remain, including dose extrapolation from animals to humans, formulation standardization, safety monitoring, and endpoint selection. Accordingly, the most realistic near-term clinical value of microbiota modulation in HS is likely to lie in risk reduction, barrier preservation, and recovery support rather than in stand-alone emergency treatment.

Clinical experience in intensive care unit (ICU) management of HS indicates that intestinal infection is a major contributor to mortality in affected patients. Early and effective gut decontamination has therefore become an important component of HS treatment protocols (71). HS can damage small-intestinal architecture, delay gastric emptying, and impair gastrointestinal function. In addition, it disrupts tight junctions in gastrointestinal epithelial cells, leading to barrier dysfunction and an increased risk of bacterial translocation into the circulation (11). Certain potentially protective taxa, including *Akkermansia muciniphila*, *Faecalibacterium prausnitzii*, *Roseburia*, and selected *Eubacterium* strains, have been proposed as indicators of a more resilient intestinal barrier. On the basis of mucosa-associated microbial profiles, personalized strategies involving prebiotics, probiotics, and dietary modulation may be developed (71).

In the emergency setting, HS management remains highly time-sensitive (72). The primary clinical priority is to reduce core body temperature as rapidly as possible, ideally within the earliest therapeutic window after collapse, while maintaining circulation and preventing secondary organ injury. Within this framework, intestinal protection may be regarded as an adjunctive strategy aimed at limiting endotoxin translocation and attenuating the inflammatory cascade (73). A pragmatic management pathway may include early cooling, volume resuscitation, electrolyte correction, monitoring of hepatic, renal, and coagulation function, and selective use of microbiota-targeted interventions in patients at high risk of gut barrier injury (74). Although the optimal timing for microbiota modulation has not been standardized, available evidence suggests that preventive administration before heat exposure or very early intervention after HS may be more effective than delayed use after multiple organ dysfunction has developed (75).

Potential clinical candidates include probiotics with documented safety profiles, synbiotics designed to enhance colonization and metabolite production, and postbiotics, which may offer more predictable composition and lower contamination risk than live bacterial products. Fecal microbiota transplantation (FMT) remains theoretically attractive but requires strict donor screening and is currently more suitable for research settings. For TCM formulations, future clinical application should depend on standardized quality control, ingredient characterization, and independent validation rather than mechanistic plausibility alone.

### 4.1 Probiotics

**Figure 2:**
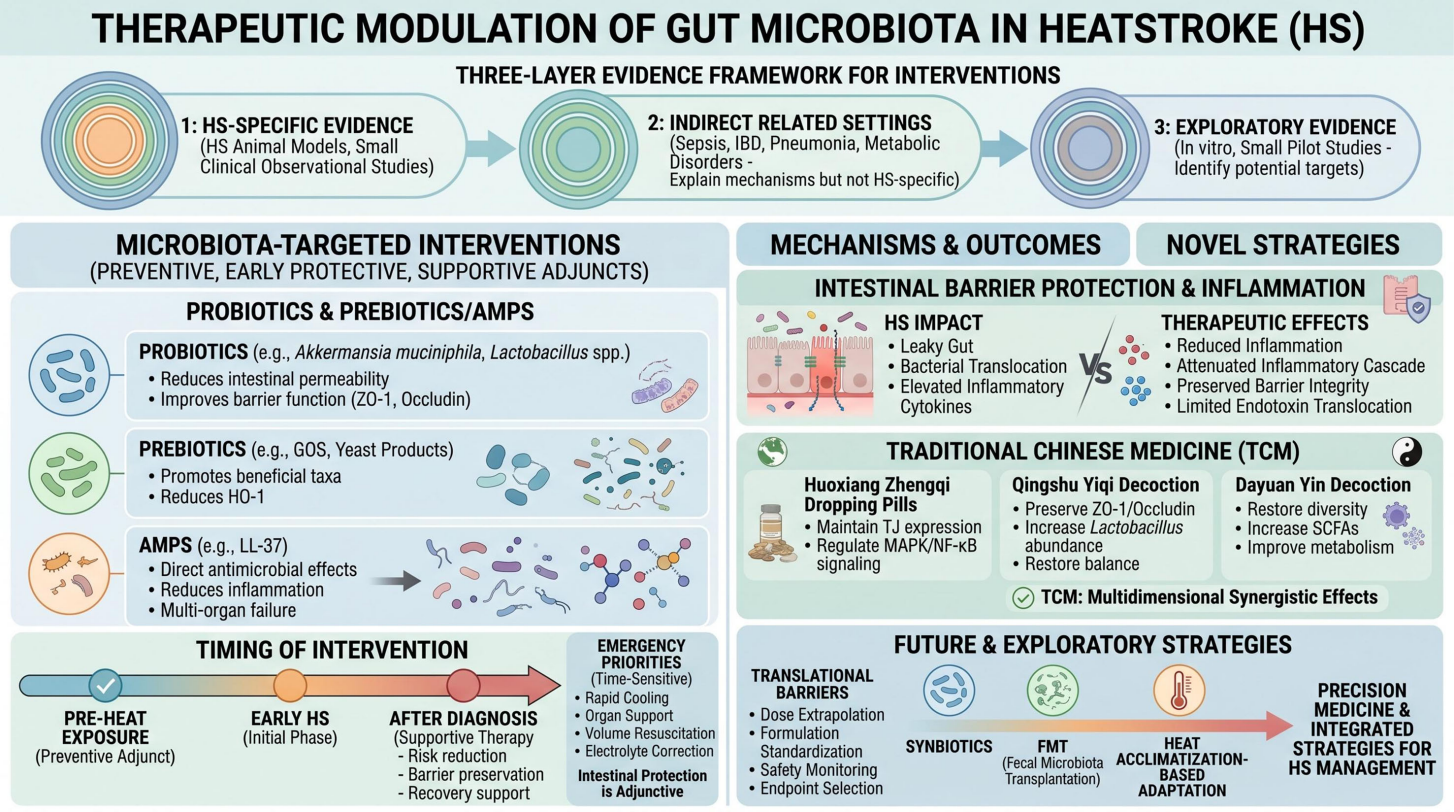
Therapeutic modulation of the gut microbiota in heat stroke: evidence framework, intervention strategies, mechanisms, and future perspectives. Microbiota-targeted interventions may serve as preventive, early protective, or supportive adjunctive strategies in HS. These approaches mainly include probiotics, prebiotics, and antimicrobial peptides. Probiotics, such as *Akkermansia muciniphila* and *Lactobacillus* spp., may reduce intestinal permeability and improve barrier function by preserving tight junction proteins, including ZO-1 and Occludin. Prebiotics, such as galactooligosaccharides and yeast-derived products, may promote beneficial microbial taxa and modulate oxidative stress-related responses. Antimicrobial peptides, such as *LL-37*, may exert direct antimicrobial effects, attenuate inflammation, and potentially reduce the risk of multiorgan injury. Mechanistically, HS can induce leaky gut, bacterial translocation, and elevated inflammatory cytokine levels. Effective microbiota modulation may alleviate inflammatory cascades, preserve intestinal barrier integrity, and limit endotoxin translocation. Traditional Chinese medicine also shows potential multidimensional synergistic effects. For example, Huoxiang Zhengqi Dropping Pills, Qingshu Yiqi Decoction, and Dayuan Yin Decoction may protect against HS-related intestinal injury by maintaining tight junction expression, regulating MAPK/NF-κB signaling, increasing *Lactobacillus* abundance, restoring microbial balance, enhancing short-chain fatty acid production, and improving metabolic homeostasis. Future studies should address key translational barriers, including dose extrapolation, formulation standardization, safety monitoring, and endpoint selection. Emerging strategies such as synbiotics, fecal microbiota transplantation, and heat acclimatization-based adaptation may further support precision medicine and integrated therapeutic approaches for HS management.

HS impairs intestinal barrier integrity and increases intestinal permeability. Probiotics can enhance intestinal barrier function by increasing the stability of epithelial tight junctions, reducing permeability, and promoting mucus secretion (76). Studies have shown that fermented yeast products protect intestinal barrier function in heat-stressed rats by modulating the gut microbiota, maintaining tight junction protein expression, preventing Paneth cell and goblet cell loss, and increasing beneficial bacteria while reducing harmful bacteria (77). *Akkermansia muciniphila* has been reported to increase the expression of the tight junction proteins occludin and ZO-1, thereby ameliorating heat stress-induced disruption of intestinal epithelial tight junction structures (78). An oral rehydration solution containing the probiotics *Lactobacillus acidophilus* NCFM, *Lactobacillus rhamnosus* GG, and *Bifidobacterium* HN019 effectively improved intestinal heat tolerance in rats (79), optimized gut microbiota composition, maintained intestinal barrier stability, and significantly attenuated HS-induced multiple organ injury, with upregulation of beneficial Lactobacillus species and downregulation of pathogenic *Streptococcus* species. The probiotic strain *Lactobacillus plantarum* MB452 improves intestinal barrier integrity by inducing the transcription of genes encoding tight junction proteins such as occludin and cingulin (80). A separate study demonstrated that *Lactobacillus plantarum* modulates tight junction protein expression in human intestinal epithelial cells *in vivo* and protects against chemically induced intestinal epithelial barrier damage *in vitro*. Duodenal administration of *Lactobacillus plantarum* in healthy volunteers significantly increased the levels of the tight junction-associated proteins zonulin and occludin (81). These findings suggest that *Lactobacillus plantarum* may enhance the stability of the intestinal tight junction complex and mitigate its disruption by cytokines, toxins, and pathogens. In addition to *Lactobacillus plantarum*, other probiotic strains within the *Lactobacillus* genus, including *Lactobacillus salivarius* (82), *Lactobacillus rhamnosus* GG (79), *Lactobacillus casei* (83), *Bacillus licheniformis* (26), and *Lactobacillus casei* (84), have also demonstrated protective effects on intestinal barrier function, potentially contributing to the amelioration of HS-associated symptoms (**Figure 2**).

Studies have confirmed that vancomycin alleviates HS symptoms by remodeling gut microbiota composition (85, 86). The use of vancomycin enemas to manage intestinal infections has also been reported in clinical practice. Probiotics are defined as live microorganisms that confer health benefits to the host when administered in adequate amounts; they enhance intestinal barrier function by maintaining gut microbiota homeostasis and modulating the intestinal immune system (87). The incidence of gastrointestinal symptoms in HS patients is 18.5%, with elderly patients and those presenting with lower Glasgow Coma Scale scores upon admission being more susceptible (88). Athletes, a high-risk group for HS, may benefit from supplementation with specific probiotics, which enhance intestinal barrier function, reduce inflammation, and improve thermotolerance and heat acclimatization through modulation of the gut microbiota, thereby exerting preventive effects against HS (71). Thermotolerance is defined here as the ability to maintain cellular and systemic integrity under heat stress conditions and to recover homeostasis after thermal exposure. Refined sugars promote bile secretion, which in turn facilitates the growth of opportunistic pathogens such as *Clostridioides difficile* and *Clostridium perfringens*. In contrast, complex carbohydrates increase the abundance of beneficial bacteria, including *Bifidobacterium* species, *Bifidobacterium longum subsp. longum*, *Bifidobacterium breve*, and *Bacteroides thetaiotaomicron*. Vegetarian diets, rich in dietary fiber, promote the production of SCFAs by the gut microbiota while lowering intestinal pH, thereby inhibiting the growth of *Escherichia coli* and other potentially pathogenic members of the *Enterobacteriaceae* family.

### 4.2 Prebiotics and antimicrobial peptides

Prebiotics can alleviate heat stress-induced intestinal barrier injury through multiple mechanisms (**Figure 2A**). Studies have shown that fermented yeast products protect intestinal barrier function in heat-stressed rats by modulating the gut microbiota, maintaining tight junction protein expression, preventing Paneth cell and goblet cell loss, and increasing beneficial bacteria while reducing harmful bacteria (77). Galacto-oligosaccharides (GOS), a well-recognized prebiotic, play an important role in protecting the intestinal barrier. Using a human colorectal adenocarcinoma (Caco-2) cell model, researchers found that GOS pretreatment prevented the upregulation of heat shock proteins induced by heat stress (40–42 °C) and reduced levels of heme oxygenase-1 (HO-1), a marker of the heat stress response, thereby mitigating heat stress-induced damage to the intestinal epithelial barrier (26).

Additionally, antimicrobial peptides (AMPs), as essential components of intestinal innate immunity, play a critical protective role in heat stress-related intestinal injury. LL-37 is the mature form of the human antimicrobial peptide cathelicidin. It exerts antimicrobial effects by selectively disrupting microbial membrane components through electrostatic interactions. Moreover, accumulating evidence indicates that LL-37 modulates innate immune responses via chemokine receptors to defend against invading microorganisms. Studies have confirmed that LL-37 ameliorates heat stress- and LPS-induced intestinal cytotoxicity, reduces intestinal damage and inflammatory responses, alleviates systemic inflammation and nitrosative stress caused by sustained hyperthermia, and significantly improves hypothermia, hypotension, vascular hyporeactivity, multiple organ dysfunction, coagulation disorders, and survival rates in HS rats (89). Notably, high expression of antimicrobial peptides in ileal samples from patients with inflammatory bowel disease is associated with better clinical outcomes, further highlighting the importance of antimicrobial peptides in intestinal barrier protection and inflammatory regulation (89).

### 4.3 FMT and postbiotics

FMT is a theoretically attractive strategy for restoring microbial diversity and functional resilience after heat-induced dysbiosis (90). In principle, FMT may replenish depleted commensals, restore metabolite balance, and promote epithelial repair. However, its use in HS remains exploratory, with no established clinical protocol (91). Important barriers include donor screening, safety monitoring, preparation standardization, and the uncertain optimal timing of administration. At present, FMT should be viewed as a research-oriented intervention rather than a routine clinical therapy for HS.

Postbiotics represent another promising direction because they may reproduce beneficial microbial functions without requiring live bacteria (92). Defined microbial metabolites or inactivated components could offer advantages in terms of safety, storage, and standardization, especially in critically ill or immunocompromised patients (93). Potential postbiotic candidates include SCFAs, bile acid derivatives, microbial peptides, and cell-wall associated components that regulate barrier function and immune signaling (94). Compared with live probiotics, postbiotics may have better manufacturing consistency and lower risk of translocation, but the available evidence in HS is still extremely limited. This area deserves further investigation.

### 4.4 Traditional Chinese medicine

With respect to intestinal barrier protection, Huoxiang Zhengqi Dropping Pills maintain the stable expression of tight junction proteins and preserve the integrity of tight junction structures between intestinal epithelial cells (**Figure 2A**). Transmission electron microscopy has confirmed that this formulation significantly attenuates heat stress-induced disruption of intestinal epithelial tight junctions, thereby preserving intestinal physical barrier function (95). These findings are consistent with broader evidence showing that heat stress compromises gut barrier integrity by disrupting epithelial tight junctions, increasing permeability, and facilitating endotoxin translocation, thereby making the intestine a central therapeutic target in HS-related injury (96).

Regarding anti-inflammatory mechanisms, Huoxiang Zhengqi Dropping Pills regulate the MAPK/NF-κB signaling pathway, inhibiting its excessive activation induced by HS, thereby alleviating local intestinal inflammation and systemic inflammatory response syndrome (SIRS) and mitigating HS-associated inflammatory injury (95, 97). This mechanistic interpretation is biologically plausible, as HS has been shown to trigger intestinal oxidative and inflammatory injury, and barrier damage can amplify systemic cytokine responses and endotoxemia (96). In terms of gut microbiota modulation, *Bushen Huatan* formula positively reshape the composition of the gut microbiota, increasing the relative abundance of beneficial bacteria such as *Lactococcus* while reducing the abundance of harmful bacteria such as *Firmicutes*, thereby contributing to the restoration of intestinal microecological balance (98). More generally, recent experimental work also supports the concept that interventions capable of improving gut-derived metabolites such as SCFAs may help protect against HS-induced organ injury and restore microbiota homeostasis (28, 96, 99, 100).

Regarding intestinal barrier protection, Qingshu Yiqi Decoction effectively maintains the expression levels of tight junction proteins (ZO-1 and occludin) and reduces levels of intestinal epithelial injury markers such as intestinal fatty acid-binding protein (**Figure 2A**). Transmission electron microscopy has demonstrated that this formulation mitigates heat stress-induced disruption of intestinal epithelial tight junctions (99). With respect to gut microbiota modulation, Qingshu Yiqi Decoction positively reshapes the gut microbiota composition and significantly increases the abundance of *Lactobacillus*, a genus with established heat stress-protective effects. 16S rRNA sequencing analysis indicates that this formulation ameliorates heat stress-induced gut dysbiosis and restores microecological balance. Network pharmacology studies have further revealed the multi-target characteristics of this formulation, providing a scientific basis for elucidating its active components and mechanisms of action.

Dayuan Yin Decoction restores gut microbiota diversity and abundance and regulates intestinal microecological balance (**Figure 2A**). Studies have shown that this formulation significantly increases intestinal SCFAs levels and improves metabolic disturbances. SCFAs, as metabolites of the gut microbiota, play important roles in maintaining intestinal barrier function and regulating immune responses (101). This point is further supported by recent heat-stress research indicating that SCFAs can attenuate heat-induced organ injury and contribute to microbiota regulation, reinforcing the host–microbiota–metabolite framework proposed here (96).

The potential of TCM in HS deserves attention, but its evidence base should be interpreted cautiously. The currently cited studies suggest that certain formulas may improve intestinal barrier integrity, reduce inflammatory markers, and modulate gut microbiota composition; however, most evidence comes from preclinical experiments or single-center clinical observations with limited sample sizes. Moreover, many TCM formulas are multi-component preparations, which creates challenges in quality control, batch consistency, pharmacokinetic interpretation, and identification of active ingredients. These features make it difficult to determine whether the observed benefit results from direct microbiota modulation, anti-inflammatory effects, improved fluid balance, or combined mechanisms.

In summary, traditional Chinese medicine exerts multidimensional synergistic effects through “barrier protection, anti-inflammatory regulation, and microbiota modulation” to maintain intestinal homeostasis during HS, offering novel intervention strategies and research directions for the prevention and treatment of HS. Future studies should further investigate the active components, dose–response relationships, and synergistic effects of combining these formulations with other probiotics or prebiotics to promote evidence-based development and clinical translation of traditional Chinese medicine in the field of HS prevention and treatment.

### 4.4 Antibiotics and other adjunctive strategies

Antibiotics are not a primary treatment for HS-related dysbiosis, and their indiscriminate use may further disrupt microbial ecology (102, 103). Nevertheless, selective antimicrobial strategies may be considered in patients with suspected bacterial translocation, severe mucosal injury, or secondary infection, provided that the potential benefits outweigh the risk of additional dysbiosis. Importantly, antibiotics should never be portrayed as a microbiota-restoring therapy; rather, they are an adjunct used in selected clinical scenarios.

Beyond antibiotics, several additional approaches deserve mention, including nutritional support, barrier-protective interventions, and targeted metabolite replacement. For example, supplementation designed to support SCFA production, bile acid homeostasis, or tryptophan metabolism may ultimately become a useful adjunct in HS management (9). These strategies remain investigational, but they fit well with the proposed host–microbiota–metabolite framework (104). The evidence for microbiota-targeted interventions in HS differs substantially across intervention types. Probiotics are the most frequently studied approach, but the majority of available data originate from preclinical models. Prebiotics, synbiotics, postbiotics, and FMT remain underexplored in HS, and their efficacy has not been established in well-designed clinical trials (90, 105–107). In addition, the optimal strain selection, dosage, treatment duration, and timing relative to heat exposure remain unknown. Therefore, current conclusions should be viewed as preliminary.

## 5 Conclusion and perspective

This review systematically summarizes the mechanisms by which the gut microbiota contributes to HS-associated distant organ injury via the gut–liver, gut–lung, and gut–brain axes. It also provides an overview of research progress on intervention strategies—including probiotics, prebiotics, antimicrobial peptides, and traditional Chinese medicine—in preserving intestinal barrier integrity, modulating gut microbiota composition, and alleviating inflammatory responses. Current evidence suggests that targeting gut microbiota regulation offers new avenues for the prevention and treatment of HS.

Several emerging microbiota-based strategies deserve future attention. First, FMT may represent a potential approach for restoring gut microbial homeostasis, but its safety and efficacy in HS have not yet been evaluated. Second, postbiotics—defined as inanimate microorganisms and/or their metabolites that confer health benefits—may offer advantages in stability and safety compared with live probiotics. Third, heat acclimatization is accompanied by dynamic changes in gut microbiota composition and function, suggesting that microbiome adaptation may contribute to improved thermotolerance. Finally, synbiotics, which combine probiotics and prebiotics, may provide synergistic benefits, but their application in HS remains largely unexplored. These strategies warrant rigorous mechanistic and translational studies before clinical use can be considered.

In summary, gut microbiota modulation holds significant promise for the prevention and treatment of HS. Moving forward, greater integration of basic and clinical research is essential to identify key microbial targets, optimize intervention protocols, and advance the development of comprehensive gut microbiota-based strategies for HS management (**Figure 2**).
